# T-shaped mesh improvisation for laparoscopic ventral mesh rectopexy: a novel technique

**DOI:** 10.1308/003588413X13511609957056f

**Published:** 2013-01

**Authors:** A Bagul, A Clarke

**Affiliations:** Poole Hospital NHS Foundation Trust, UK

## Background

Laparoscopic ventral mesh rectopexy using a composite mesh is a technique gaining more recognition for management of pelvic floor disorders such as full thickness rectal prolapse, obstructive defecation symptoms and vaginal vault prolapse. A recent Cochrane review concluded that laparoscopic rectopexy results in fewer post-operative complications and an earlier discharge^1^ over open methods.^2^ We describe a novel technique for preparation of the mesh.

## Technique

**Figure 1:**
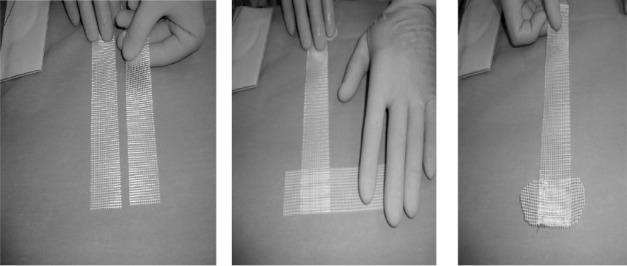
Construction of T-shaped mesh

Two longitudinal pieces of the standard 3cm x 20cm strip of polypropylene or polyester mesh are used; the second piece is placed at right angles to prepare a T shape. The second piece is stitched with four sutures, forming a T shape (Fig 1).

## Discussion

This prepared mesh is cheaper than pre-packed/shaped meshes. The T shape allows better sitting in the pelvis. It improves area for fixation to the anterior rectum and offers better support.
